# Identification of BAG3 target proteins in anaplastic thyroid cancer cells by proteomic analysis

**DOI:** 10.18632/oncotarget.23858

**Published:** 2018-01-03

**Authors:** Francesca Galdiero, Anna Maria Bello, Anna Spina, Anna Capiluongo, Sophie Liuu, Margot De Marco, Alessandra Rosati, Mario Capunzo, Maria Napolitano, Emilia Vuttariello, Mario Monaco, Daniela Califano, Maria Caterina Turco, Gennaro Chiappetta, Joëlle Vinh, Giovanni Chiappetta

**Affiliations:** ^1^ Functional Genomic Unit, Istituto Nazionale Tumori–IRCCS–Fondazione G. Pascale, Napoli, Italia; ^2^ ESPCI ParisTech, Spectrométrie de Masse Biologique et Protéomique (SMBP), USR3149 CNRS, Paris, France; ^3^ Biouniversa s.r.l., University of Salerno, Fisciano, Italy; ^4^ Department of Medicine, Surgery and Dentistry “Scuola Medica Salernitana”, University of Salerno, Baronissi (SA), Italy; ^5^ “SS. Giovanni di Dio e Ruggi d'Aragona-Schola Medica Salernitana”, University of Salerno Hospital, Salerno, Italy

**Keywords:** BAG3, anaplastic thyroid cancer, SILAC, CAV1, SERPINB2

## Abstract

BAG3 protein is an apoptosis inhibitor and is highly expressed in Anaplastic Thyroid Cancer. We investigated the entire set of proteins modulated by BAG3 silencing in the human anaplastic thyroid 8505C cancer cells by using the Stable-Isotope Labeling by Amino acids in Cell culture strategy combined with mass spectrometry analysis. By this approach we identified 37 up-regulated and 54 down-regulated proteins in BAG3-silenced cells. Many of these proteins are reportedly involved in tumor progression, invasiveness and resistance to therapies. We focused our attention on an oncogenic protein, CAV1, and a tumor suppressor protein, SERPINB2, that had not previously been reported to be modulated by BAG3. Their expression levels in BAG3-silenced cells were confirmed by qRT-PCR and western blot analyses, disclosing two novel targets of BAG3 pro-tumor activity. We also examined the dataset of proteins obtained by the quantitative proteomics analysis using two tools, Downstream Effect Analysis and Upstream Regulator Analysis of the Ingenuity Pathways Analysis software. Our analyses confirm the association of the proteome profile observed in BAG3-silenced cells with an increase in cell survival and a decrease in cell proliferation and invasion, and highlight the possible involvement of four tumor suppressor miRNAs and TP53/63 proteins in BAG3 activity.

## INTRODUCTION

Anaplastic Thyroid Cancer (ATC), accounting for 1–2% of all thyroid cancers, is the most aggressive form of thyroid cancer, with a median survival of 5 months, and is characterized by the accumulation of several oncogenic alterations [1]. This rare thyroid tumor does not respond to any chemotherapeutic regimen or radioiodine treatment thus representing a candidate target for innovative therapies, such as molecular targeted therapy [2].

Bcl2-associated athanogene 3 (BAG3) belongs to BAG family of molecular cochaperones, that interact with the ATPase domain of the heat shock protein 70 (HSP70) through the structural domain known as BAG domain [3, 4]. In addition, BAG3 protein contains a WW domain, a proline-rich region (PXXP), and two conserved IPV (Ile-Pro-Val) motifs, that can also mediate binding to signaling proteins. BAG3 protein is an anti-apoptotic factor that sustains cell survival in several tumor types [5] and is involved in different cellular processes including autophagy, cell stress response, proliferation, migration and Epithelial-Mesenchymal Transition (EMT). BAG3 expression is induced by stress stimuli in normal cells types, while is constitutive in several human cancer cells (leukemia, myeloma, neuroblastoma, pancreas, colon, glioblastoma) [6]. We previously reported that BAG3 protein is specifically expressed in thyroid carcinomas and not in normal thyroid tissue and that its down-modulation results in enhancing apoptosis and decreasing cell survival in human thyroid carcinoma cells [7].

More recently, we reported that BAG3 down-modulation inhibits ATC proliferation *in vitro* and sensitizes cells to apoptosis *in vivo*. Moreover, we found that BAG3 interacts with BRAF [8], a strong MAPK pathway activator and the most frequently mutated human oncogene in the kinase superfamily [9]. BAG3 sustains BRAF protein intracellular levels by inhibiting HSP70-mediated delivery of BRAF to the proteasome [8].

Given the BAG3 key role in regulating major molecular pathways in ATC, the aim of this study was to investigate the entire set of BAG3-regulated protein partners by a quantitative proteomics approach. We used the Stable-Isotope Labeling by Amino acids in Cell culture (SILAC) [10] strategy combined with mass spectrometry analysis to decipher the BAG3 proteome. Protein expression data from *BAG3*-silenced cells compared to control cells allowed us to identify candidate targets of BAG3-mediated regulation in anaplastic thyroid cancer.

## RESULTS

### Quantitative proteomics analysis of *BAG3*-silenced 8505C cells

Wild type 8505C cells were grown in heavy media while si*BAG3* and si*Scrambled* treated cells were grown in light media. Control cells were respectively pooled in equal amounts with si*BAG3-* and si*Scrambled-*transfected cells and protein extracts were trypsin digested and analyzed by mass spectrometry as beyond described. For each condition, samples were analyzed in 3 biological replicates (3 independent transfections) and each biological replicate was analyzed in 3 technical replicates (3 independent LC-MSMS analyses).

**Figure 1:**
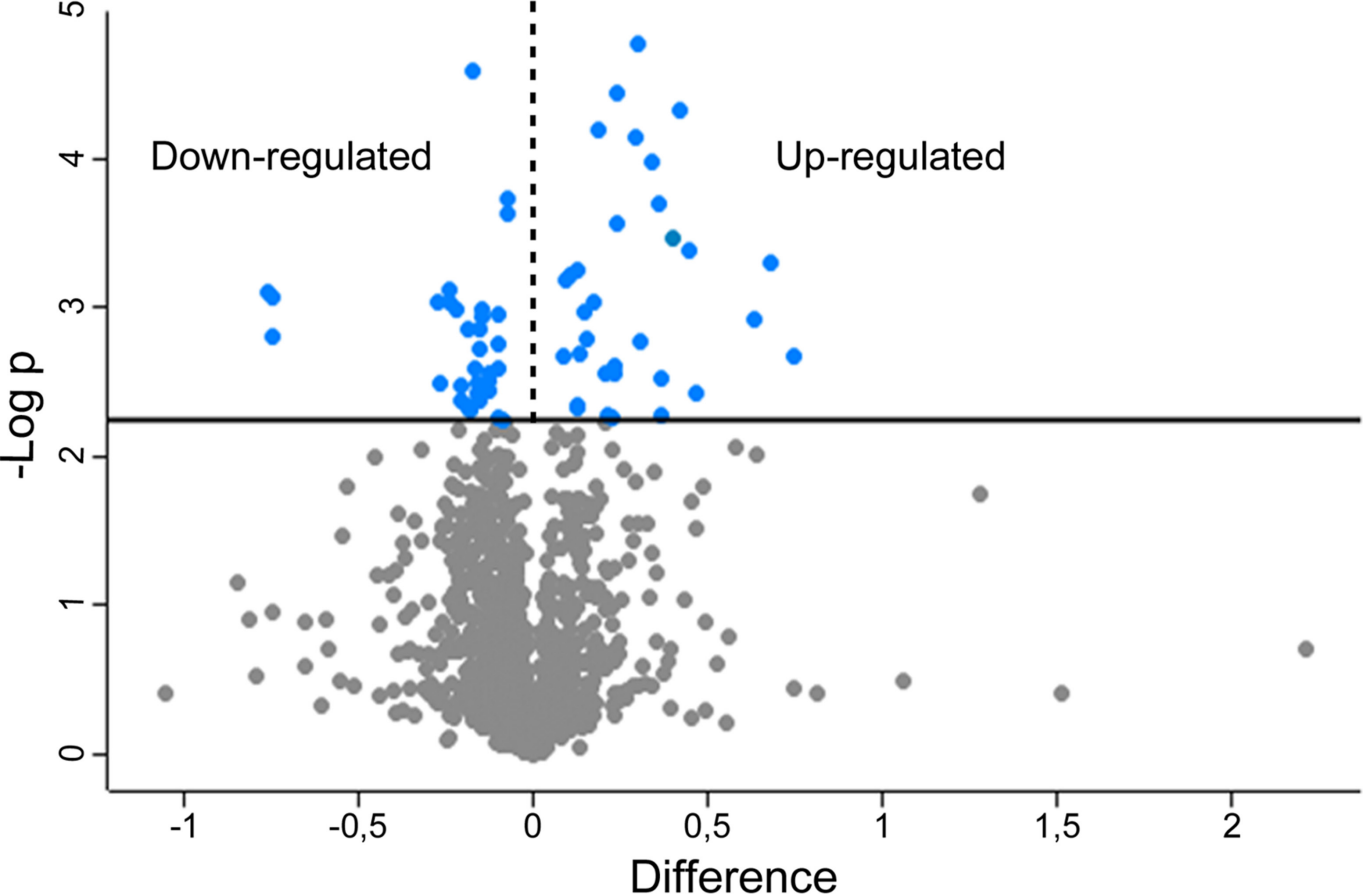
Volcano plot obtained from SILAC-based quantitative proteomics analysis Blue dots represent proteins exhibiting significative fold changes.

MS analysis allowed performing relative quantification of 1167 proteins (Supplementary Table 1). To find proteins that significantly changed expression profiles, data were mined with the software Perseus 1.5.2.6. The protein ratios obtained from the samples control *vs* si*Scrambled* were compared to the protein ratios obtained by the samples control *vs* si*BAG*3 building a “volcano plot” (Figure 1). This statistical tool allows to compare the protein expression profiles of si*Scrambled*- with the si*BAG3-*transfected cells calculating the difference between the ratios (expressed in log2) of any given protein in the two sets of samples. The calculated differences are used to perform a *t*-test.

By this approach 37 up-regulated and 54 down-regulated proteins were identified in si*BAG3*-transfected cells (Supplementary Table 2) choosing a FDR threshold of 0.07. Large part of these proteins is already reported in literature for their roles in tumor progression, invasiveness and resistance to treatments. Accordingly to the role of BAG3 in cancer proliferation and apoptosis [7, 8], we found up-regulation of 5 pro-apoptotic proteins and down-regulation of 3 anti-apoptotic proteins in *BAG3*-silenced cells. Moreover, the up-regulation of 6 anti-apoptotic proteins suggests that 8505C cells developed compensatory mechanisms to face the *BAG3* silencing (Table 1).

**Table 1:** Proteins associated to apoptosis

Pvalue (-log10)	Protein IDs	Protein names	Gene names	References
**PRO APOPTOTIC (Up-regulated)**				
3.1	Q9NYF8	Bcl-2-associated transcription factor 1	BCLAF1	[11]
3	Q07021	Complement component 1 Q subcomponent-binding protein	C1QBP	[12]
2.9	P25705	ATP synthase subunit alpha, mitochondrial	ATP5A1	[13]
2.8	Q9NQC3	Reticulon-4	RTN4	[14]
2.4	O95816	BAG family molecular chaperone regulator 2	BAG2	[15]
**ANTI APOPTOTIC (Down-regulated)**				
AIC	Q9NX55	Huntingtin-interacting protein K	HYPK	[16]
AIC	B4E0Y9	Serine/threonine-protein kinase MST4	MST4	[17]
AIC	Q96CS3	FAS-associated factor 2	FAF2	[18]
**ANTI APOPTOTIC (Up-regulated)**				
2.8	P52926	High mobility group protein HMGI-C	HMGA2	[19]
3	P05141	ADP/ATP translocase 2	SLC25A5	[20]
3.1	P38646	Stress-70 protein, mitochondrial	HSPA9	[21]
2.4	B4DT31	Far upstream element-binding protein 1	FUBP1	[22]
2.8	Q86UE4	Protein LYRIC	MTDH	[23]
AIC	O95394	Phosphoacetylglucosamine mutase	PGM3	[24]

In the table are reported the p-values (-log10) associated to the observed fold changes of proteins levels. It is interesting to underline that no pro-apoptotic proteins are observed in *BAG3*-silenced 8505C cells. AIC: “Absent In Controls”.

**Figure 2:**
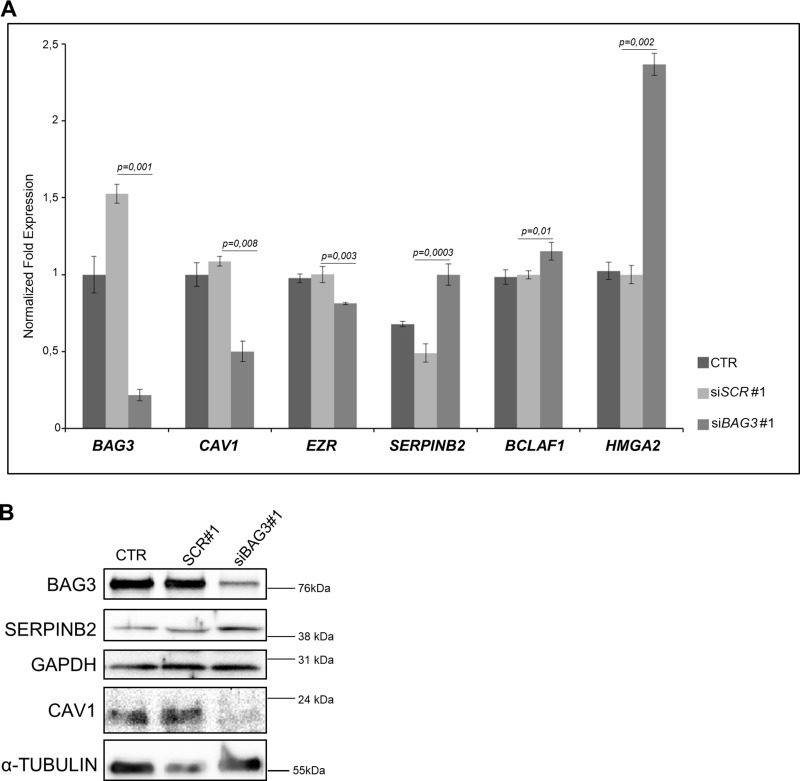
(**A**) Representative qRT-PCR analysis of *BAG3*, *CAV1, EZR, SERPINB2, BCLAF1* and *HMGA2* mRNA expression in 8505C ATC cells transfected with scrambled (si*SCR*#1) or *BAG3*-specific siRNA (si*BAG3*#1) (grown in Light SILAC Media) compared to non-transfected 8505C cells (grown in Heavy SILAC media). Data were represented as relative expression on *GAPDH*. (**B**) Representative Western Blot analysis of BAG3, SERPINB2 and CAV1 protein expression in 8505C ATC cells transfected with scrambled (si*SCR*#1) or *BAG3*-specific siRNA (si*BAG3*#1) (grown in Light SILAC Media) compared to non-transfected 8505C cells (grown in Heavy SILAC media). α-TUBULIN and GAPDH were used as loading control. These transfected cells were used for Mass Spectrometry analysis.

Proteomics data were validated with orthogonal methods monitoring expression profiles of some BAG3-modulated targets. qRT-PCR analysis confirmed *BAG3* silencing efficiency and quantitative proteomics data for *CAV1* and *EZR* down-regulation, *SERPINB2*, *BCLAF1* and *HMGA2* up-regulation. (Figure 2A).

We focused our attention on: CAV1, a 22 kDa protein considered as an indicator of thyroid carcinoma progression [25] and *serpinB2* (PAI2), a Plasminogen Activator Inhibitor that can reportedly act as a suppressor of tumor growth and metastasis formation [26]. Western blot analyses (Figure 2B) confirmed proteomics data, showing that in *BAG3*-silenced cells, CAV1 and *serpinB2* (PAI2) levels were down- and up-regulated, respectively. We tested another *BAG3* specific siRNA in 8505C (Supplementary Figure 1A–1B) and CAL-62 ATC cell lines (Supplementary Figure 2) confirming the previous results obtained about *CAV1* and *SERPINB2* modulation.

### Apoptosis evaluation

**Figure 3:**
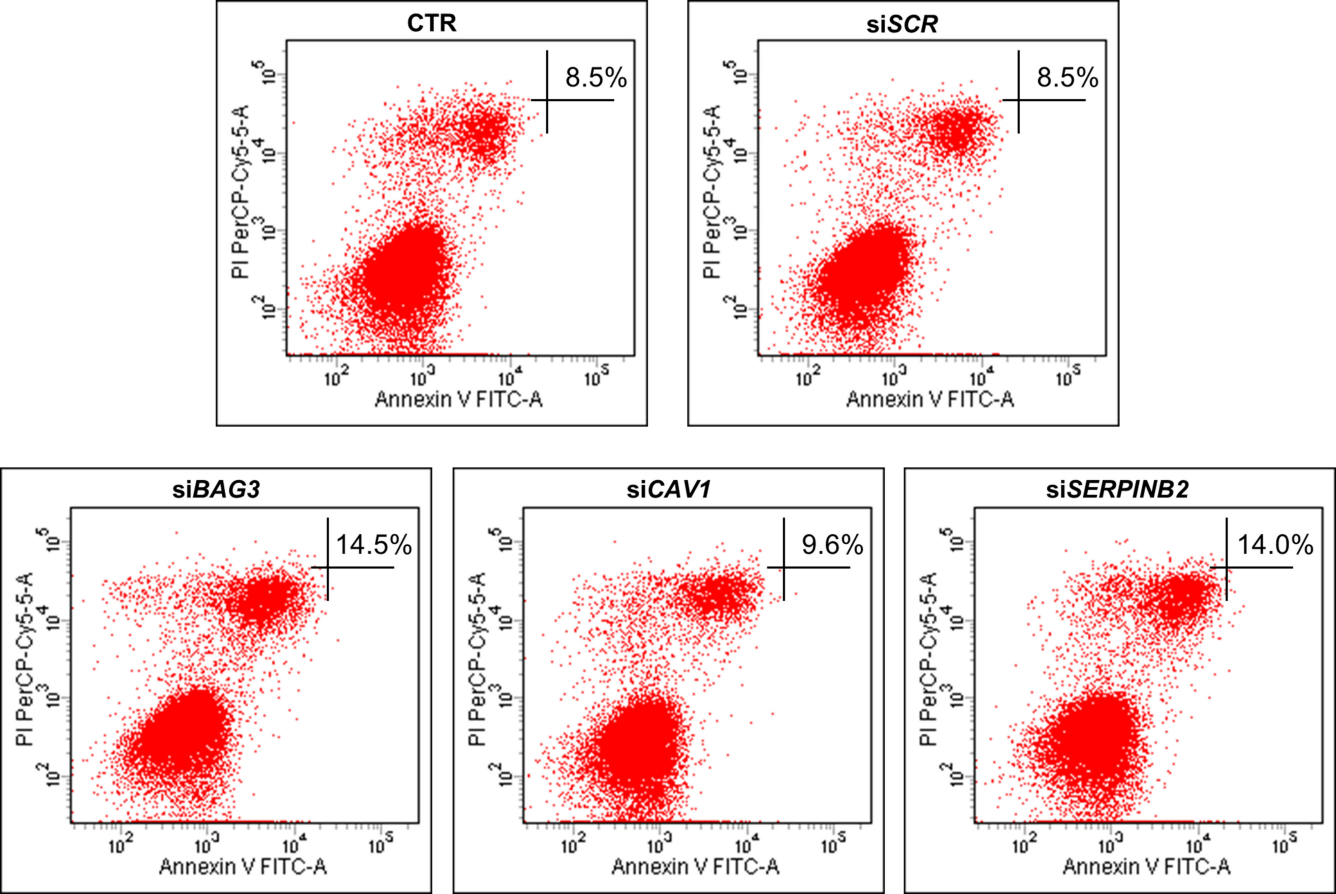
Flow cytometric detection of apoptosis in the 8505C cell line Representative figures showing population of viable (Annexin V- PI-), early apoptotic (Annexin V+ PI-), late apoptotic (Annexin V+ PI+) and necrotic (Annexin V- PI+) cells in the cells treated with si*BAG3*, si*CAV*1 and si*SERPINB2* (lower panels) compared to control or scrambled treated cells (upper panels).

BAG3 is well reported to inhibit apoptosis in ATC cells [7, 8]. The roles of CAV1 and SERPINB2 in regulating apoptosis is unclear to date. To elucidate CAV1 and SERPINB2 effects on apoptosis, we evaluated by flow cytometry the percentage on Annexin-V positive cells upon silencing of *BAG3*, *CAV*1 and *SERPINB2* in 8505C cells. The percentage of Annexin-V positive cells underwent a marked increase in si*BAG3* and si*SERPINB2* transfected cells, as compared to control cells. Percentage of late apoptotic cells in si*BAG3*, si*CAV1* and si*SERPINB2* treated cells were 14,5%, 9,6% and 14%, respectively (Figure 3). *BAG3*, *CAV1* and *SERPINB2* silencing efficiency was assessed by RT-qPCR (Supplementary Figure 3).

Taken together, these data confirm the well-known role of BAG3 as apoptosis inhibitor and suggest a similar role for SERPINB2.

### Pathways analysis

**Figure 4:**
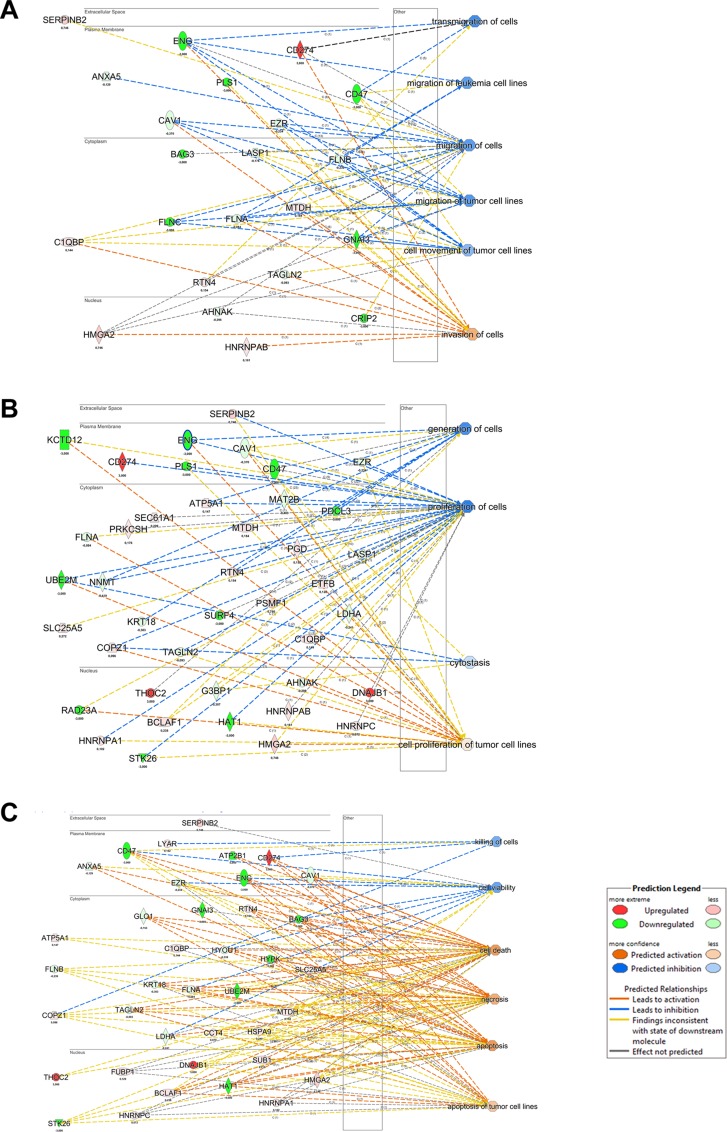
IPA Downstream Effect Analysis obtained from quantitative proteomics analysis of *BAG*3 silenced ATC cells (**A**) Causal effects of protein modulation and the reduced cell migration. (**B**) Causal effects of protein modulation and the reduced cell proliferation. (**C**) Causal effects of protein modulation and the increase apoptosis.

The list of proteins exhibiting expression profile variations was submitted to IPA software using the Core Analysis function. Considering the *Downstream Effect Analysis* (DEA) of IPA, “Cell Death and Survival” functions resulted increased, while the “Cell Movement” and “Cell growth and proliferation” functions (Figure 4A–4C) decreased. These data are in agreement with previous studies where it was showed that BAG3 silencing increased apoptosis in 8505C cells and in xenografts [8].

Furthermore, the IPA/DEA results showed also that, aside the global decrease of invasiveness and growth, *BAG3-*silenced cells adapted to this perturbation modifying the expression levels of proteins involved in compensatory molecular mechanisms.

**Figure 5:**
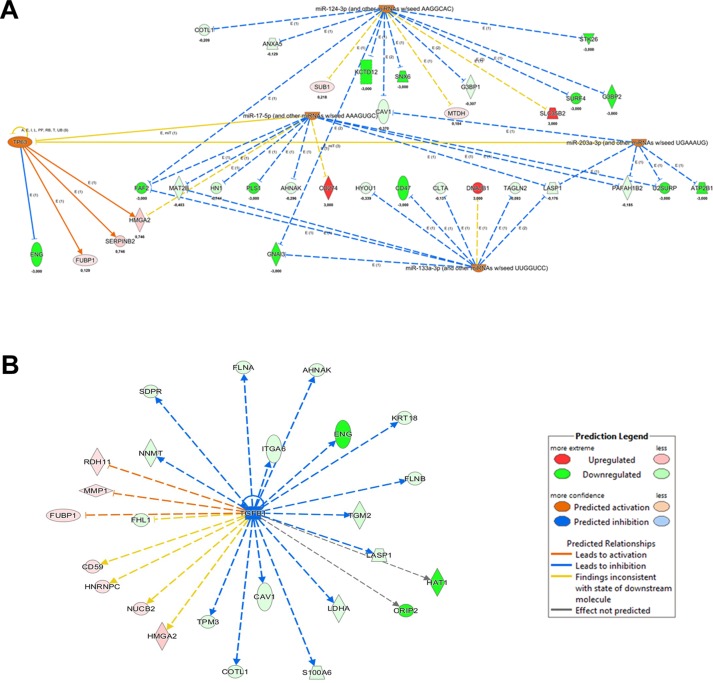
IPA Upstream Regulator Analysis obtained from quantitative proteomics analysis of *BAG3* silenced ATC cells (**A**) Regulators predicted up-regulated and their causal links with the modulated protein in *BAG3* silenced ATC cells are showed. (**B**) Upstream regulator TGFβ1 is predicted down-regulated in agreement with a decreased cell proliferation.

*Upstream Regulator Analysis* (URA) is another tool of IPA allowing to predict the upstream molecules (transcription factor, microRNA) that could have a causal role in the observed proteome profiling. Submitting to IPA/URA our dataset, we obtained the enrichment of 5 precursors that we can distinguish in two clusters (Figure 5A). The first one is characterized by the predicted activation of 4 tumor suppressor miRNAs (miR-133a-3p, miR-203a-3p, miR-17-5p, miR-124-3p) [27–30]. These miRNAs are associated to the expression control of proteins already evidenced by DEA to be related to increase of cell death and decrease of invasiveness.

The second is the transcription factor TP63, whose levels are predicted to be increased in a compensatory adaptation of 8505C cells to *BAG3* silencing. TP63 is a member of TP53 family, whose oncogene/onco-suppressor features have been long debated [31]. Finally, the most significant upstream factor predicted to be inhibited is the growth factor *TGFβ1*, confirming the relation between *BAG3* silencing and tumor growth repression (Figure 5B).

## DISCUSSION

BAG3 appears to influence cell survival by interacting with different molecular partners, thus activating multiple pathways. A first demonstrated mechanism of BAG3 anti-apoptotic activity is mediated by its role as a co-chaperone in protein delivery to the proteasome. For example, BAG3 protects IKK-γ from proteasome delivery and this results in sustained NF-kB activation and cell survival [5]. We can speculate that, through its binding to HSP70, BAG3 might also positively or negatively modulate folding of other apoptosis-regulating proteins. A different mechanism has been observed in glioblastoma cells, in which BAG3 is over-expressed and retains BAX protein in the cytosol, preventing its mitochondrial translocation. In this tumor model BAG3 could be used as a potential target for therapy [32]. In this study we found that *BAG3* silencing led to up-regulation of 5 pro-apoptotic proteins. Among these, BCLAF1 can induce apoptosis by its transcriptional repressor activity [11]. In tumor cells, BCLAF1, a nuclear protein, can be sequestered into the cytoplasm by the antiapoptotic BCL2 family members, blocking its pro-apoptotic activity. The increased levels of BCLAF1 upon *BAG3* silencing suggests that in ATC cells BAG3 might interfere with degradation of this protein assisted by proteasome. The fact that BAG3 and BCLAF1 are interacting protein could sustain this hypothesis [33].

Otherwise, BAG3 can interfere with apoptosis signaling protecting anti-apoptotic proteins from degradation. However, the anti-apoptotic proteins found down-regulated in our study do not appear to be BAG3 interactors. Among the anti-apoptotic proteins regulated by BAG3, we found up-regulation of HMGA2 in *siBAG3*- treated cells. In different cancer models, HMGA2 act as an apoptosis inhibitor inducing BCL2 expression and exerting an opposite effect on the Caspase activity [34, 35].

*BAG3* silencing in cancer cells reduces invasion [6] and our proteomics study in 8505C cell line and IPA data analysis are in agreement with this activity. Indeed we found decreased levels of 6 proteins involved in cell migration. Among these EZRIN is a BAG3 partner [33] that could escape from degradation in ATC through the mechanism of client stabilization cooperated by HSP70.

We found that *BAG3* silencing induces the down-modulation of CAV1. Caveolin proteins are the main integral proteins of caveolae, that are key regulators of signal transduction, extracellular matrix organization and cell migration, and are involved also in metabolic alterations in cancer cells [36]. CAV1 is highly expressed in thyroid anaplastic carcinomas, and in papillary carcinomas it is considered as an indicator of tumor progression [25]. The concomitant decreased levels of Cavin1 (PTRF) protein, necessary for caveolae formation, reinforces the idea of an alteration of these ATC oncogenic structures associated to BAG3. On the other hand, among genes whose expression was up-modulated in *BAG3*-silenced cells there is the Plasminogen Activator Inhibitor type 2 SERPINB2. Two forms of Plasminogen activator inhibitor are known, respectively SERPINE1 (PAI1) and SERPINB2 (PAI2). Despite shared serpin function, high tumor levels of SERPINE1 promote tumor progression, whereas high levels of SERPINB2 appear to decrease tumor growth and metastasis [26]. This divergence was related to their competition to bind urokinase plasminogen activation system, that led to cell migration induction.

It was already demonstrated the existence of a link between the protein levels of CAV1, SERPINE1 and TGFβ. Indeed, SERPINE1 gene induction is mediated by Reactive Oxygen Species (ROS) after TGFβ exposition. CAV1 is necessary to this activation; indeed, CAV1 deficiency suppresses the expression of SERPINE1 induced by TGFβ. Unfortunately, SERPINE1 expression data are missing in our proteomics study, but from literature SERPINE1 levels can be predicted to be down-modulated in these conditions [37].

Moreover, it is interesting to underline that the URA data analysis by IPA predicted a reduced activity of TGFβ pathway in *BAG3*-silenced 8505C cells. High levels of this cytokine are secreted by thyroid tumor cells [38]. A previous study showed that TGFβ induces BAG3 over-expression and that SERPINE1 over-expression is independent of BAG3 in HK2 cells [39]. All these findings lead us to hypothesize the existence of a missing link between BAG3, TGFβ, Caveolin proteins, Serpins and cell migration that needs to be elucidated.

Further investigation should be performed to verify if *BAG3* silencing influences TGFβ secretion or if it can sensitize/desensitize cells to this cytokine. Moreover, in our opinion, the possible correlation between the decrease of CAV1/SERPINE1 and the increase of SERPINB2 levels is worthy of investigation. These findings will be useful to understand the relations between BAG3 and cell migration and a potential enhancing of the “protective” effects of SERPINB2.

It was supposed that, besides its activity in apoptosis and cell migration, BAG3 could sustain tumors also at different levels [6]. *BAG3* silencing in ATC cells is associated to the down-regulation of 12 proteins implicated in oncogenic mechanisms such as proliferation, hypoxia, detoxification, DNA repair, mitochondria metabolism and drug resistance (Supplementary Table 2).

The wideness of our proteomics study allowed to detect the development of adaptive mechanisms to escape from apoptosis after *BAG3* silencing. Indeed, we found increased levels of 6 proteins with a key role in anti-apoptotic mechanism (Table 1). In future, it will be interesting to elucidate the possible cross-talk mechanisms between these proteins and *BAG3* silencing as adaptive response in ATC.

In conclusion, in this study, by a proteomic approach, we analyzed protein pathways upon *BAG3* silencing. Among these, we found variations of proteins involved in apoptosis, cell migration, proliferation, hypoxia, detoxification, DNA repair, mitochondrial metabolism and drug resistance. This study is a proof of concept predicting multiple roles of BAG3 in regulating major cellular pathways and puts the basis for future works.

## MATERIALS AND METHODS

### Cells and SILAC labeling

The human thyroid anaplastic carcinoma cell line 8505C was cultured as monolayers according to the instruction of the provider (European Collection of Cell Cultures, Salisbury, UK). The culture media DMEM (GE Healthcare Life Science, Buckinghamshire, UK) was supplemented with 10% fetal bovine serum (FBS) (GE Healthcare HyClone), 100 U/ml penicillin and streptomycin (LONZA, Verviers, Belgium), 2 mM L-Glutamine (LONZA) and 1% Non-Essential Amino Acids (Gibco, Carlsbad, CA, USA). Cells were maintained at 37°C in a humidified atmosphere with 5% CO_2_ for some cell doublings and then transferred in SILAC medium. 8505C cell line was adapted to the SILAC media as described earlier [40]. SILAC labeling and culturing was performed using PIERCE SILAC Protein Quantitation KIT (Thermo Scientific, MA, USA) according to manufacturer’ instructions. SILAC medium consisted of DMEM medium with 4 mM L-Glutamine, 110 mg/l Sodium Pyruvate, 4.5 g/l Glucose and without L-Arginine and L-Lysine. “Light” DMEM medium was supplemented with 10% v/v dialyzed fetal bovine serum, 0.1 mg/ml L-Lysine-2HCl and 0.1 mg/ml L-Arginine-HCl. “Heavy” DMEM medium was supplemented with 10% v/v dialyzed fetal bovine serum, 0.1 mg/ml ^13^C_6_ L-Lysine-2HCl and 0.1 mg/ml ^13^C_6_^15^N_4_ L-Arginine. DMEM media containing dissolved amino acids were sterile-filtered using 0.22 μm filter and stored at 4°C protected from light. After six cell doublings, the incorporation of stable isotopes was higher than 99% as determined by mass spectrometry analysis.

The human thyroid anaplastic carcinoma cell line CAL-62 was cultured as monolayers. The culture media DMEM (GE Healthcare Life Science) was supplemented with 10% fetal bovine serum (FBS) (GE Healthcare HyClone), 100 U/ml penicillin and streptomycin (LONZA), 2 mM L-Glutamine (LONZA). Cells were maintained at 37°C in a humidified atmosphere with 5% CO_2_.

### Small interfering RNA (siRNA) and transfection

For SILAC proteomic analyses, 8505C cells growth in “light” SILAC media were transfected with siRNA specific for *Bag3* (si*BAG3#1*: 5′-AAGGUUCAGACCAUCUUGGAA-3′) or scrambled siRNA (si*SCR*#1: 5′-CAGUCGCGUUUG CGACUGG-3′) (synthesized by GE Healthcare Dharmacon, Buckinghamshire, UK) and used at a final concentration of 200 nM. 8505C cells growth in “light” media were plated at 30-40% confluence and transfected with siRNAs using Transfectin (Bio-Rad, Hercules, CA, USA) according to manufacturer's instructions. 8505C cells growth in “heavy” SILAC medium were used as non-transfected control. Transfections were carried out in triplicate. Cells were harvested 72 h after transfection and equal amount of “light” (si*BAG3* or si*Scrambled* transfected cells) and “heavy” cells were pooled together.

Other small-interfering RNA used were: si*BAG3*#2 (Cat. No. SR306333), si*SERPINB2* (Cat. No. SR303346), si*CAV1* (Cat. No. SR300603) (synthesized by OriGene Technologies, Rockville, MD, USA), used according to the manufacturer's recommendations. The scrambled nonsense siRNA (si*SCR*#2) (Universal scrambled negative control siRNA duplex, Cat. No. SR30004), which has no homology to any known gene, was used as control. Cell transfection with siRNA oligonucleotides was performed using Transfectin (Bio-Rad) according to manufacturer's recommendations and as above described.

### RNA extraction and quantitative Real Time qRT-PCR

RNA extraction was performed using the Qiagen RNeasy Mini Kit according to the manufacturer's instructions. RNA was eluted in 30 μl of RNase free water. The RNA concentration was measured with Eppendorf BioSpectrometer^®^ (Eppendorf AG, Germany). cDNA was synthesized using the Quantitect ReverseTrascription Kit (Qiagen, Valencia, CA, USA) according to the manufacturer's instructions. qRT-PCR analysis was carried out using the iTaq™ Universal SYBR® Green Supermix (Bio-Rad), according to manufacturer's instructions. All primers for PCR amplification were designed through the Primer3 tool (http://biotools.umassmed.edu/bioapps/primer3_www.cgi). Primers sequences and amplicon size, are listed in Supplementary Table 3.

### Protein extraction and western blot analysis

Cells were harvested and lysed in a buffer containing 50 mM Tris/HCl pH 7.5, 150 mM NaCl, 1 mM EDTA, 1% NP40 added with protease and phosphatase inhibitors (Roche, Switzerland). Lysates were centrifuged at 13 200 rpm at 4°C and the proteins recovered from the supernatant were quantified by Bradford assay. 30 μg of total protein were separated on 12% (wt/vol) SDS/PAGE gels under denaturing conditions and transferred to nitrocellulose membrane (GE Healthcare Life Science) with a 0.4 μm porosity. The nitrocellulose membrane was incubated 1 h with blocking solution of TBS–BSA containing 25 mM Tris pH 7.4, 200 mM NaCl, 5% BSA and phenol red and subsequently incubated overnight with primary antibody. Proteins electrophoresed on 12% (wt/vol) SDS/PAGE gel were hybridized with anti-BAG3 rabbit polyclonal antibody TOS-2 (BIOUNIVERSA s.r.l., Fisciano, SA, Italy) and anti-PAI2 rabbit polyclonal antibody H-70 (Santa Cruz Biotechnology, Texas, USA) and anti-CAV1 mouse monoclonal antibody 7C8 (Santa Cruz Biotechnology). Anti-α-Tubulin mouse monoclonal antibody (Sigma-Aldrich, Saint Louis, MO, USA) and anti-GAPDH rabbit polyclonal antibody (TREVIGEN, Gaithersburg, MD, USA) were used as loading control. Immunoreactivity was detected by incubation with horseradish peroxidase-conjugated secondary antibody (Bio-Rad) and enhanced chemiluminescence reagents (SuperSignal West Dura Extended Duration Substrate, Pierce, Thermo Fisher Scientific) following manufacturer’ protocol. Bands were digitally visualized using ChemiDoc XRS+ (Bio-Rad) imager and images were captured using Image Lab^TM^ Software (Bio-Rad).

### Evaluation of apoptosis by DNA-flow cytometry

Apoptosis evaluation was performed using FITC Annexin V Apoptosis Detection Kit I (Catalog No. 556547, BD Pharmigen, Sparks, Maryland, USA). Briefly, 8505C cells, transfected with specific siRNA targeting *BAG3*, *CAV1*, *SERPINB2* and scrambled siRNA used as control, were harvested 48 h after transfections. According to manufacturer's instructions, cells were washed twice with cold PBS and then resuspended in 1X Binding Buffer at a concentration of 1 × 106 cells/ml. 100 μl of the solution (1 × 10^5^ cells) were incubated with 5 μl of FITC Annexin V and 5 μl of Propidium Iodide (PI) and incubated 15 min in the dark. 400 μl of 1X Binding Buffer was added to each tube. A minimum of 20 000 events for each sample was collected. Data acquisition was performed using BD FACSCanto II Flow Cytometer, and data were analyzed using BD FacsDiva 6.1.3 (BD Biosciences).

### Protein digestion

Aliquots of 2 × 10^6^ cells were harvested by sonication in 600 μl of denaturing buffer (Urea 6 M, Tris 100 mM pH 8.1, 1% octyl β-glucopyranoside, protease inhibitor Complete (Sigma Aldrich)) supplemented with 10 mM of dithiothreitol (DTT). After sonication, samples were incubated for 2 h at 37°C under stirring. Cysteine alkylation was carried out in 55 mM iodoacetammide for 30 min at room temperature, under stirring in the dark. Insoluble pellet was removed and proteins were precipitated by adding 600 μl of 20% trichloroacetic acid (TCA) followed by centrifugation for 1 h at 4°C at 13 000 rpm. For digestion, protein pellets were then resuspended in 100 μl of 50 mM ammonium bicarbonate (Fluka, Sigma-Aldrich) with 2 μg of bovine sequencing-grade trypsin (Roche) over night at 37°C under stirring.

### LC-MS/MS

5 μl of each sample were fractionated on a capillary reverse phase column (nano C18 Dionex Acclaim PepMap100, particle size 3 μm, 75 μm i.d. x 50 cm) at constant flow rate of 220 nl/min, with a gradient of 2% to 50% of Buffer B in Buffer A for 180 min (Buffer A: 98% water, 2% LC-MS grade acetonitrile (ACN) (Fisher Chemical, Thermo Fisher Scientific), 0.1% formic acid; Buffer B: 90% ACN, 10% water, 0.1% formic acid). Liquid Chromatography (LC) was directly coupled to a Q Exactive mass spectrometer (Thermo Fisher Scientific). MS experiments consisted of a survey MS scan (400 to 2 000 m/z; resolution 70 000) followed by an MS/MS analysis of the most 10 intense precursors, with a dynamic exclusion of 30 s of the previously fragmented precursors. AGC target for MS was set at 3e^6^, maximum injection time of 100 msec. MS/MS experiments were performed in HCD mode. Precursor ions were selected with an isolation window of 2 m/z. AGC target for MS/MS was set at 3e^6^, maximum injection time of 120 msec, resolution 17 500. Each sample was analyzed in triplicate runs.

### Data analysis

Database analysis was performed using MaxQuant (version 1.3.0.5) with Swiss-Prot database (version 2015_05). Methionine oxidation and cysteine carbamidomethylation were selected as variable modifications. First search error tolerance was 20 ppm while main search error tolerance was set to 6 ppm. MS/MS search was performed with 20 ppm of error tolerance. Protein identification was performed with a False Discovery Rate (FDR) of 1%. Protein relative quantification was performed calculating the mass spectrometry intensity ratios of heavy (^13^C_6_^15^N_4_ L-Arginine and ^13^C_6_ L-Lysine) and light forms of peptides. Each protein was quantified with at least 2 Unique/Razor peptides. Data mining was performed using Perseus 1.5.2. The quantified proteins in the sample si*BAG3* vs Control were compared to the quantified protein of si*Scrambled* vs Control sample using the Volcano plot tool. Missing values were treated as follow: when a protein hit was not detected in 2 biological replicates it is considered absent in the given condition. No imputations were made when a protein hit was missing in one biological sample.

### Ingenuity pathway analysis

For ingenuity pathway analysis (IPA) (http://www.ingenuity.com) the accession numbers from Uniprot database were generated from MaxQuant. IPA generates the functional analysis based on proteins fold changes of a given datasets showing a rank-ordered list of pathways whose activities are most likely affected. Fisher's exact test was used to calculate a *p* value determining the probability that each biological function assigned to the data set is due to change alone. Protein dataset was created from Perseus 1.5.2, exporting protein hits exhibiting significative fold changes with a FDR threshold of 0.07. Proteins considered absent in a given conditions were added to the dataset imputing an arbitrary fold change (log2) of +/− 3 respectively if up or down regulated in *BAG3* silenced cells.

## SUPPLEMENTARY MATERIALS FIGURES AND TABLES






