# Supplementary material for: Identification of BAG3 target proteins in anaplastic thyroid cancer cells by proteomic analysis
Source: Oncotarget. 2018 Jan 3;9(8):8016–26. doi: 10.18632/oncotarget.23858 (PMC5814278; doi:10.18632/oncotarget.23858)
Supplement: Supplementary file 1 [file oncotarget-09-8016-s001.pdf]

Identification of BAG3 target proteins in anaplastic thyroid cancer cells by proteomic analysis

SUPPLEMENTARY MATERIALS

Supplementary Table 1: List of proteins quantified by SILAC based quantitative proteomics analysis. Fold changes are expressed in log2. See_Supplementary_Table_1

Supplementary Table 2: List of proteins exhibiting significative expression fold changes. Red typed proteins are up-regulated, blue typed proteins are down-regulated. See_Supplementary_Table_2

SUPPLEMENTARY TABLE 2 REFERENCES

1. Croucher DR, Saunders DN, Lobov S, Ranson M. Revisiting the biological roles of PAI2 (SERPINB2) in cancer. Nat Rev Cancer. 2008; 8:535–545.
2. Mansoori B, Mohammadi A, Shirjang S, Baradaran B. HMGI-C suppressing induces P53/caspase9 axis to regulate apoptosis in breast adenocarcinoma cells. Cell Cycle. 2016; 15:2585–2592.
3. Bauer MK, Schubert A, Rocks O, Grimm S. Adenine nucleotide translocase-1, a component of the permeability transition pore, can dominantly induce apoptosis. J Cell Biol. 1999; 147:1493–1502.
4. Liu T, Krysiak K, Shirai CL, Kim S, Shao J, Ndonwi M, Walter MJ. Knockdown of HSPA9 induces TP53-dependent apoptosis in human hematopoietic progenitor cells. PLoS One. 2017; 12:e0170470.
5. Kasof GM, Goyal L, White E. Btf, a novel death-promoting transcriptional repressor that interacts with Bcl-2-related proteins. Mol Cell Biol. 1999; 19:4390–4404.
6. Schommer C, Debernardi JM, Bresso EG, Rodriguez RE, Palatnik JF. Repression of cell proliferation by miR319-regulated TCP4. Mol Plant. 2014; 7:1533–1544.
7. Pavon-Eternod M, Gomes S, Geslain R, Dai Q, Rosner MR, Pan T. tRNA over-expression in breast cancer and functional consequences. Nucleic Acids Res. 2009; 37:7268–7280.
8. Meng X, Brachova P, Yang S, Xiong Z, Zhang Y, Thiel KW, Leslie KK. Knockdown of MTDH sensitizes endometrial cancer cells to cell death induction by death receptor ligand TRAIL and HDAC inhibitor LBH589 co-treatment. PLoS One. 2011; 6:e20920.
9. Jiang X, Sun Q, Li H, Li K, Ren X. The role of phosphoglycerate mutase 1 in tumor aerobic glycolysis and its potential therapeutic implications. Int J Cancer. 2014; 135:1991–1996.
10. He Y, Brown MA, Rothnagel JA, Saunders NA, Smith R. Roles of heterogeneous nuclear ribonucleoproteins A and B in cell proliferation. J Cell Sci. 2005; 118:3173–3183.
11. Wang HQ, Zhang HY, Hao FJ, Meng X, Guan Y, Du ZX. Induction of BAG2 protein during proteasome inhibitor-induced apoptosis in thyroid carcinoma cells. Br J Pharmacol. 2008; 155:655–660.
12. Watari A, Yutsudo M. Multi-functional gene ASY/Nogo/RTN-X/RTN4:apoptosis, tumor suppression, and inhibition of neuronal regeneration. Apoptosis. 2003; 8:5–9.
13. Singh S, Khar A. Differential gene expression during apoptosis induced by a serum factor: role of mitochondrial F0-F1 ATP synthase complex. Apoptosis. 2005; 10:1469–1482.
14. Itahana K, Zhang Y. Mitochondrial p32 is a critical mediator of ARF-induced apoptosis. Cancer Cell. 2008; 13:542–553.
15. Lee CH, Jeong SJ, Yun SM, Kim JH, Lee HJ, Ahn KS, Zhu S, Chen CY, Kim SH. Down-regulation of phosphoglucomutase 3 mediates sulforaphane-induced cell death in LNCaP prostate cancer cells. Proteome Sci. 2010; 8:67.
16. Xue G, Hao LQ, Ding FX, Mei Q, Huang JJ, Fu CG, Yan HL, Sun SH. Expression of annexin a5 is associated with higher tumor stage and poor prognosis in colorectal adenocarcinomas. J Clin Gastroenterol. 2009; 43:831–837.
17. Smith CM, Haucke V, McCluskey A, Robinson PJ, Chircop M. Inhibition of clathrin by pitstop 2 activates the spindle assembly checkpoint and induces cell death in dividing HeLa cancer cells. Mol Cancer. 2013; 12:4.
18. Creighton DJ, Zheng ZB, Holewinski R, Hamilton DS, Eiseman JL. Glyoxalase I inhibitors in cancer chemotherapy. Biochem Soc Trans. 2003; 31:1378–1382.

19. Nishikawa R, Goto Y, Sakamoto S, Chiyomaru T, Enokida H, Kojima S, Kinoshita T, Yamamoto N, Nakagawa M, Naya Y, Ichikawa T, Seki N. Tumor-suppressive microRNA-218 inhibits cancer cell migration and invasion via targeting of LASP1 in prostate cancer. Cancer Sci. 2014; 105:802–811.
20. Chang JW, Zuhl AM, Speers AE, Niessen S, Brown SJ, Mulvihill MM, Fan YC, Spicer TP, Southern M, Scampavia L, Fernandez-Vega V, Dix MM, Cameron MD, et al. Selective inhibitor of platelet-activating factor acetylhydrolases 1b2 and 1b3 that impairs cancer cell survival. ACS Chem Biol. 2015, 10:925–932.
21. Meng Y, Lu Z, Yu S, Zhang Q, Ma Y, Chen J. Ezrin promotes invasion and metastasis of pancreatic cancer cells. J Transl Med. 2010; 8:61.
22. Miao P, Sheng S, Sun X, Liu J, Huang G. Lactate dehydrogenase A in cancer: a promising target for diagnosis and therapy. IUBMB Life. 2013; 65:904–910.
23. Bonora M, Wieckowsk MR, Chinopoulos C, Kepp O, Kroemer G, Galluzzi L, Pinton P. Molecular mechanisms of cell death: central implication of ATP synthase in mitochondrial permeability transition. Oncogene. 2015; 34:1608.
24. Stojadinovic A, Hooke JA, Shriver CD, Nissan A, Kovatich AJ, Kao TC, Ponniah S, Peoples GE, Moroni M. HYOU1/Orp150 expression in breast cancer. Med Sci Monit. 2007; 13:BR231–239.
25. Nassar ZD, Hill MM, Parton RG, Parat MO. Caveola-forming proteins caveolin-1 and PTRF in prostate cancer. Nat Rev Urol. 2013; 10:529–536.
26. Paskas S, Jankovic J, Marecko I, Isic Dencic T, Tatic S, Cvejic D, Savin S. Caveolin-1 expression in papillary thyroid carcinoma: correlation with clinicopathological parameters and BRAF mutation status. Otolaryngol Head Neck Surg. 2014; 150:201–209.
27. Peng H, Dara L, Li TW, Zheng Y, Yang H, Tomasi ML, Tomasi I, Giordano P, Mato JM, Lu SC. MAT2B-GIT1 interplay activates MEK1/ERK 1 and 2 to induce growth in human liver and colon cancer. Hepatology. 2013; 57:2299–2313.
28. Xu J, Moatamed F, Caldwell JS, Walker JR, Kraiem Z, Taki K, Brent GA, Hershman JM. Enhanced expression of nicotinamide N-methyltransferase in human papillary thyroid carcinoma cells. J Clin Endocrinol Metab. 2003; 88:4990–4996.
29. Traba J, Del Arco A, Duchen MR, Szabadkai G, Satrustegui J. SCaMC-1 promotes cancer cell survival by desensitizing mitochondrial permeability transition via ATP/ADP-mediated matrix Ca(2+) buffering. Cell Death Differ. 2012; 19:650–660.
30. Itkonen HM, Engedal N, Babaie E, Luhr M, Guldvik IJ, Minner S, Hohloch J, Tsourlakis MC, Schlomm T, Mills IG. UAP1 is overexpressed in prostate cancer and is protective against inhibitors of N-linked glycosylation. Oncogene. 2015;34:3744–3750.
31. Choudhury KR, Raychaudhuri S, Bhattacharyya NP. Identification of HYPK-interacting proteins reveals involvement of HYPK in regulating cell growth, cell cycle, unfolded protein response and cell death. PLoS One. 2012; 7:e51415.
32. Srinivasan S, Meyer RD, Lugo R, Rahimi N. Identification of PDCL3 as a novel chaperone protein involved in the generation of functional VEGF receptor 2. J Biol Chem. 2013; 288:23171–23181.
33. Xia Y, Yan LH, Huang B, Liu M, Liu X, Huang C. Pathogenic mutation of UBQLN2 impairs its interaction with UBXD8 and disrupts endoplasmic reticulum-associated protein degradation. J Neurochem. 2014; 129:99–106.
34. Rosen LS, Gordon MS, Robert F, Matei DE. Endoglin for targeted cancer treatment. Curr Oncol Rep. 2014; 16:365.
35. Zhang Y, Yao J, Huan L, Lian J, Bao C, Li Y, Ge C, Li J, Yao M, Liang L, He X. GNAI3 inhibits tumor cell migration and invasion and is post-transcriptionally regulated by miR-222 in hepatocellular carcinoma. Cancer Lett. 2015; 356:978–984.
36. Sotgia F, Whitaker-Menezes D, Martinez-Outschoorn UE, Salem AF, Tsirigos A, Lamb R, Sneddon S, Hulit J, Howell A, Lisanti MP. Mitochondria "fuel" breast cancer metabolism: fifteen markers of mitochondrial biogenesis label epithelial cancer cells, but are excluded from adjacent stromal cells. Cell Cycle. 2012; 11:4390–4401.
37. Lo PH, Ko JM, Yu ZY, Law S, Wang LD, Li JL, Srivastava G, Tsao SW, Stanbridge EJ, Lung ML. The LIM domain protein, CRIP2, promotes apoptosis in esophageal squamous cell carcinoma. Cancer Lett. 2012; 316:39–45.
38. Xiong W, Matheson CJ, Xu M, Backos DS, Mills TS, Salian-Mehta S, Kiseljak-Vassiliades K, Reigan P, Wierman ME. Structure-Based Screen Identification of a Mammalian Ste20-like Kinase 4 (MST4) Inhibitor with Therapeutic Potential for Pituitary Tumors. Mol Cancer Ther. 2016; 15:412–420.

Supplementary Table 3: Characteristics of primers pairs

Acronym	Gene Name	Acc. No.	Primer Forward (5′-3′)	Primer Reverse (5′-3′)	Amplicon size
BAG3	BCL2 associated athanogene 3	NM_004281.3	CTCAGCCAGATAAACAGTGTGG	GTCAGAGGCAGCTGGAGACT	101 bp
BCLAF1	BCL2 associated transcription factor 1	NM_014739.2<br>NM_001077440.1<br>NM_001301038.1	GGAAACTGCAAAGACTGGGA	GAAGCCTCTTTATCCCTGGTATT	99 bp
CAV1	Caveolin 1	NM_001172897.1	GCGACCCTAAACACCTCAAC	TGCCGTCAAAACTGTGTGTC	90 bp
EZR	Ezrin	NM_003379.4<br>NM_001111077.1	GGAACAGACCTTTGGCTTGG	TGTTCCTGATTTCACTCCAAGG	106 bp
GAPDH	Glyceraldehyde-3-phosphate	NM_002046.4	CCCTTCATTGACCTCAACTACATG	TGGGATTTCCATTGATGACAAGC	109 bp
HMGA2	High mobility group A2	NM_003483.4<br>NM_003484.1	GCTCAGAAGAGAGGAC	GGTCTCTTAGGAGAGGGCTCA	77 bp
SERPINB2	Serpin family B member 2	NM_001143818	GTGGGTTCATGCAGCAGATC	AGAGCGGAAGGATGAATGGA	92 bp

A

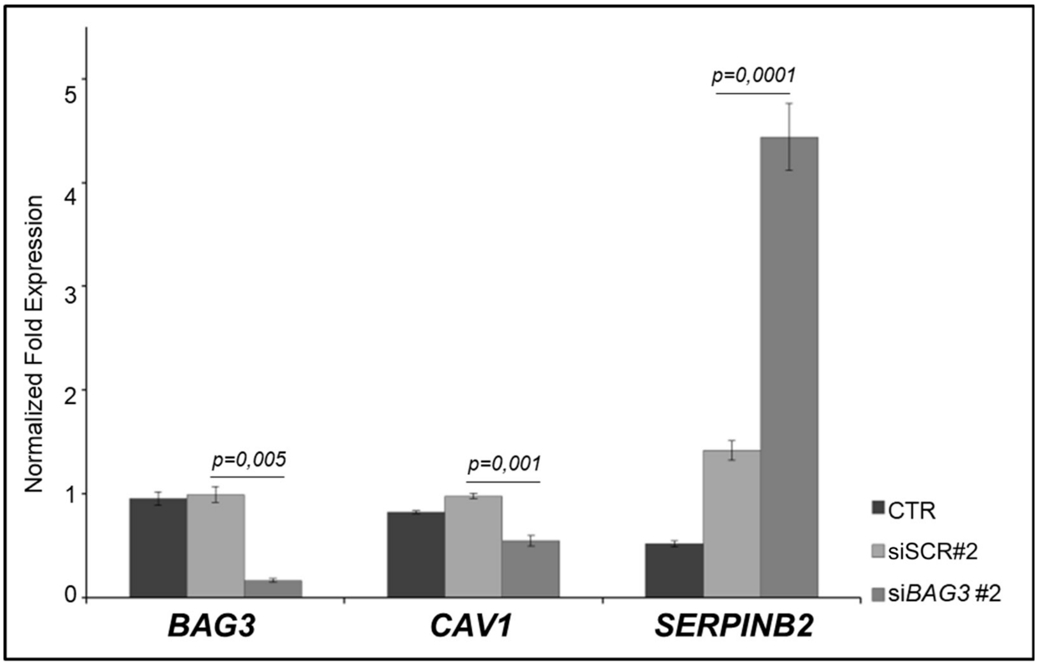

B

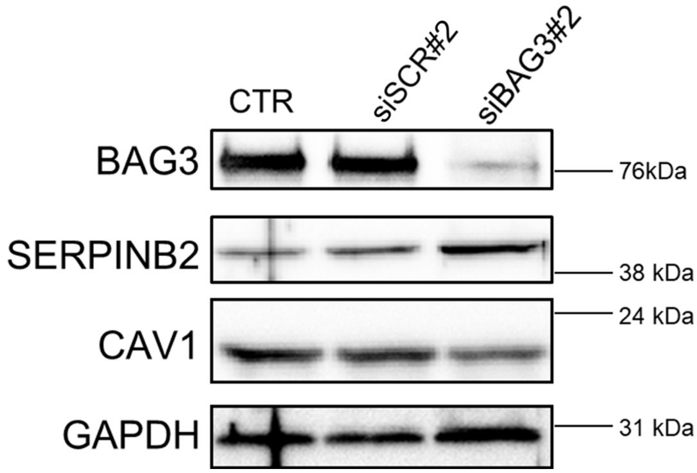

Supplementary Figure 1: (**A**) Representative qRT-PCR analysis of *BAG3*, *CAV1* and *SERPINB2* mRNA expression in 8505C ATC cells transfected with scrambled (*siSCR*#2) or *BAG3*-specific siRNA (si*BAG3*#2) compared to non-transfected 8505C cells. Data were represented as relative expression on *GAPDH*. (**B**) Representative Western Blot analysis of BAG3, SERPINB2 and CAV1 protein expression in 8505C ATC cells transfected with scrambled (*siSCR*#2) or *BAG3*-specific siRNA (*siBAG3*#2) compared to non-transfected 8505C cells. GAPDH was used as loading control.

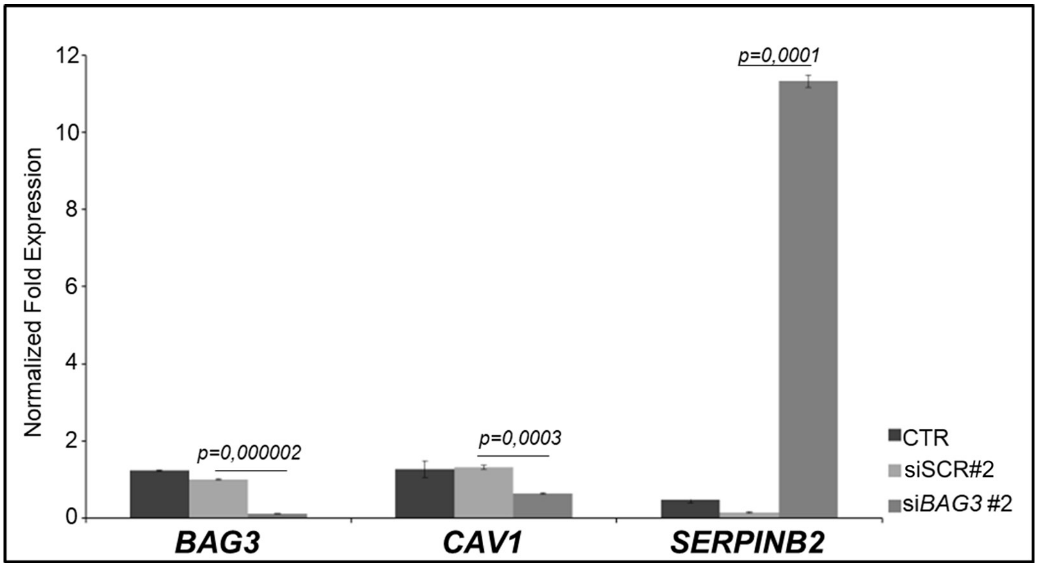

Supplementary Figure 2: Representative qRT-PCR analysis of *BAG3*, *CAV1* and *SERPINB2* mRNA expression in CAL-62 ATC cells transfected with scrambled (si*SCR*#2) or *BAG3*-specific siRNA (si*BAG3*#2) compared to non-transfected 8505C cells. Data were represented as relative expression on *GAPDH*.

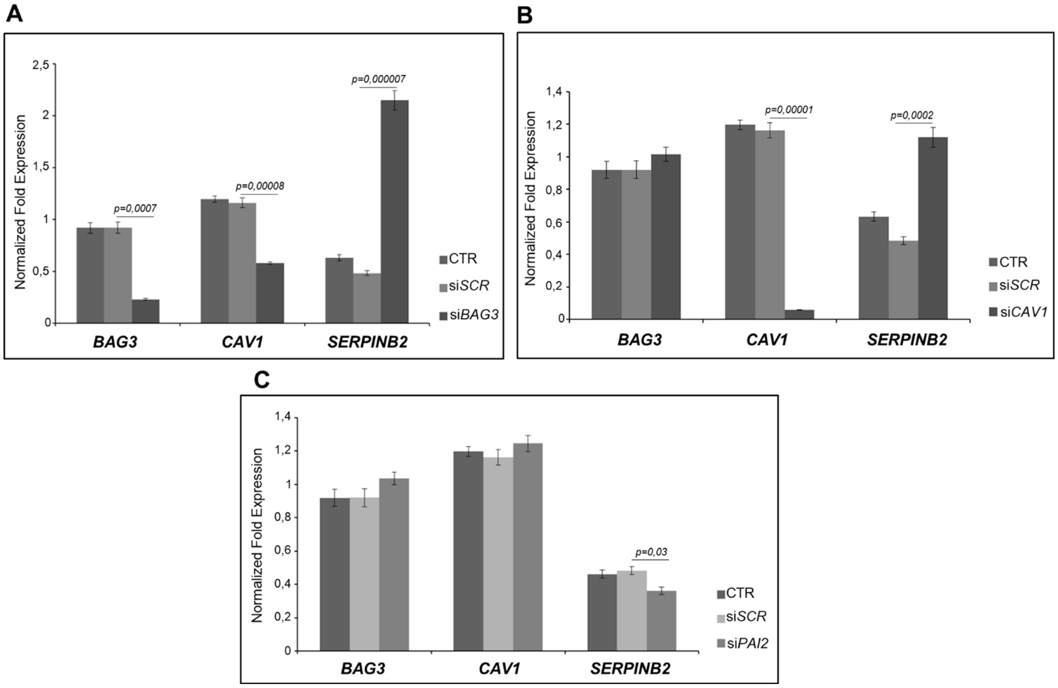

Supplementary Figure 3: Representative qRT-PCR analysis of *BAG3*, *CAV1* and *SERPINB2* mRNA expression in 8505C cells transfected with scrambled (si*SCR*) or (**A**) *BAG3*-specific siRNA (si*BAG3*), (**B**) *CAV1*-specific siRNA (si*CAV1*) and (**C**) *SERPINB2*-specific siRNA (si*SERPINB2*) , compared to non-transfected 8505C cells. Data were represented as relative expression on *GAPDH*.
